# Association between maternal lifestyle factors and low birth weight in preterm and term births: a case-control study

**DOI:** 10.1186/s12978-020-00932-9

**Published:** 2020-06-11

**Authors:** Chuhao Xi, Min Luo, Tian Wang, Yingxiang Wang, Songbai Wang, Lan Guo, Ciyong Lu

**Affiliations:** 1 Department of Medical Statistics and Epidemiology, School of Public Health, Sun Yat-sen University, Guangzhou, 510080 People’s Republic of China; 2 Guangdong Provincial Key Laboratory of Food, Nutrition and Health, Sun Yat-sen University, Guangzhou, 510080 People’s Republic of China; 3 Maternity and Child Health Care Hospital of Guangdong, Jiangmen, China

**Keywords:** Low birth weight, Lifestyle factors, Physical activity, Passive smoking, Case-control study

## Abstract

**Background:**

It has been reported that lifestyle factors may affect birth weight; however, few studies have explored the association between lifestyle factors and low birth weight in preterm and term births in China. The objective of this study was to explore the effect of lifestyle on low birth weight in preterm and term births.

**Methods:**

This case-control study was conducted in fourteen hospitals in Jiangmen, Guangdong Province. Data were collected from August 2015 to May 2016 using a standard questionnaire. Data were analysed using logistic regression.

**Results:**

Women who delivered preterm and were physically active (1–3 times per week and ≥ 4 times per week) had reduced odds of having low birth weight babies (aOR = 0.584, 95%CI = 0.394–0.867 and, aOR = 0.516, 95%CI = 0.355–0.752, respectively). Pregnant women who had insufficient gestational weight gain had increased odds of having low birth weight babies (aOR = 2.272, 95%CI = 1.626–3.176). Women exposed to passive smoking had an increased risk of delivering low birth weight infants (aOR = 1.404, 95%CI = 1.057–1.864). Insufficient gestational weight gain and excessive gestational weight gain were both significantly associated with low birth weight (aOR = 1.484, 95%CI = 1.103–1.998 and aOR = 0.369, 95%CI = 0.236–0.577, respectively) for term deliveries. In addition, parity, history of low birth weight, antenatal care and gestational hypertension were significantly associated with the likelihood of low birth weight.

**Conclusion:**

Pregnant women without exercise contraindications should remain physically active. Pregnant women should be aware of the negative effects of smoke and be aware of strategies to protect themselves from passive smoke exposure. Hospitals should inform pregnant women of the importance appropriate gestational weight gain. These recommendations should be put into practice to decrease the prevalence of low birth weight infants.

## Plain English summary

Low birth weight is a serious condition in developing countries. Although lifestyle factors may affect low birth weight, few studies have explored the association between lifestyle factors and low birth weight in preterm and term births.

This study was conducted in fourteen randomly selected hospitals in Jiangmen, Guangdong Province, China. Data were collected from August 2015 to May 2016 using a questionnaire and medical records. A total of 294 preterm low birth weight infants, 289 term low birth weight infants and 1381 controls were selected.

In the preterm low birth weight group, physical activity and insufficient gestational weight gain were associated with low birth weight. Meanwhile, in the term low birth weight group, insufficient gestational weight gain and excessive gestational weight gain were both significantly associated with low birth weight. In addition, in the term low birth weight group, women who experienced passive smoking had an increased risk of delivering low birth weight infants.

In conclusion; findings from this study may help hospitals develop proper, immediate and sustainable measures to improve maternal and child health.

## Background

Low birth weight (LBW) is defined as less than 2500 g at birth, irrespective of the gestation age [1]. Babies born with LBW have been shown to have diminished cognitive development [2], and evidence now suggests that LBW babies are at an increased risk of chronic diseases later in life, including high blood pressure, non-insulin dependent diabetes mellitus, coronary heart disease and stroke [3, 4]. According to the United Nations Children’s Fund (UNICEF) report, LBW contributes to 40–60% of newborn mortality globally [2]. Moreover, the World Health Organization (WHO) reports LBW as a serious disease that has become an important risk factor for increased disease burden worldwide [5].

The average prevalence rate of LBW is 15% worldwide [6, 7]. In mainland China, an epidemiological survey in 2011 showed that the incidence of LBW was 6.1% [8], which is higher than the incidence of 5.87% reported in 1998. However, another study in northwest China reported a decrease in prevalence of LBW deliveries from 4.4% in 2010 to 3.6% in 2013 [9].

Many lifestyle factors have been associated with birth weight [10–13]. Previous studies have shown that smoking [11] or exposure to second hand smoke [12], drinking [10], physical activity [14], and variations in energy intake may lead to higher rates of LBW. However, some studies examining the relationship between lifestyle factors and LBW have shown conflicting results [15–17]. Studies have shown that most lifestyle factors have different effects on preterm and term infant’s birth weight [12, 18–21]; nevertheless, there is a paucity of studies exploring the association of lifestyle factors with LBW in term and preterm babies separately. Moreover, because race and cultural backgrounds differ regionally, data from one region [12, 22, 23] are difficult to extrapolate to other areas. To date, there is little data from developing countries, particularly ones as diverse and complex as China. This fact prompted us to examine risk factors for outcomes related to preterm and term LBW. This study was conducted to investigate the impact of lifestyle factors on LBW in preterm and term births.

## Methods

### Study design

This case-control study was conducted in fourteen hospitals in Jiangmen, Guangdong Province. A stratified sampling method was adopted according to region, and then purposive selection was performed based on number of deliveries. Hospitals were chosen to ensure that there were at least two hospitals in each region. The cases and controls in this study were recruited from the same hospitals from August 2015 to May 2016. Informed consent was obtained from each woman.

### Sample size and subject selection

The sample size was determined using the proportion difference approach with the assumption of a 95% confidence level (Z_α/2_ = 1.96), 80% power (Z_β_ = 0.84), a control to case ratio of 1:2 (r = 2), an odds ratio to be detected ≥2 and an exposure of 3.5% for the control group [4, 24, 25]. Only deliveries > 28 gestational weeks were included. Cases and controls that did not meet selection criteria were excluded, and the final sample size was 1964 (583 cases and 1381 controls).

Cases were divided into two groups: preterm LBW and term LBW. The selection criteria for the first subgroup were singleton births with a birth weight < 2500 g and gestational age < 37 weeks. The selection criteria for the second subgroup were singleton births with a birth weight < 2500 g and gestational age ≥ 37 weeks. The exclusion criteria were as follows: 1) twins or multiple births, 2) congenital anomalies and stillbirths, and 3) birth weight ≥ 4000 g. A total of 294 preterm LBW infants and 289 term LBW infants were selected.

For each case, two pregnant women who delivered at the same hospital and their newborns were selected as the controls. The control group inclusion criteria were as follows: 1) singleton births, 2) delivery time close to that of the case and 3) a birth weight ≥ 2500 g. The exclusion criteria were the same as those for the cases. Finally, 1381 controls were selected.

### Data collection procedure

Data were collected through a questionnaire and prospective case enrolment. The questionnaire was designed by local clinical experts and research team after group consultation and review. An obstetric nurse with a public health background facilitated questionnaire completion in the postnatal ward. High-risk lifestyle factors were only assessed in the questionnaire. The questionnaire included sections focused on demographics, lifestyle factors, and other potential LBW-related factors. Maternal demographic characteristics included age, height, pre-pregnancy weight, level of education, average income per person in the family, and marital status. Lifestyle factors included cigarette smoking, passive smoking, drinking, physical activity, coffee or tea drinking and gestational weight. The interviewees were questioned about whether they smoked and how many cigarettes were smoked per day [26]. Passive smoking (second-hand smoking), according to the definition of the WHO, was defined as indirect exposure to smoke exhaled more than 15 min at least 1 day a week during pregnancy. The pregnant women self-reported their passive smoke exposure status and daily duration of exposure during pregnancy at home, in the workplace and in public places [27]. Regarding drinking, the women were asked whether they drank alcohol and if so, the quantity consumed (e.g., number of cans, glasses, bottles) on a typical occasion for each of four types of alcoholic beverages (beer, wine/champagne, spirits/liqueurs, and fortified wines) [28]. Physical activity questions were designed to capture the frequency, duration and intensity of activity, and were based on questions and responses from previous studies [29]. Physical activity in our study was defined as moderate-intensity exercise, such as taking a walk, jogging and traditional Chinese Taiji boxing [30, 31]. The number of cups per day of coffee and tea consumed in a week was asked. Self-reported gestational weight gain was used to evaluate the energy intake. Based on the body mass index (BMI) and the 2009 guide from the Institute of Medicine (IOM) [32], normal gestational weight gain was defined as follows: when the patient’s BMI was < 18.5, 18.5 ≤ BMI ≤ 23.9, 24.0 ≤ BMI ≤ 27.9 and BMI ≥ 28, weight gain should be 12.5-18 kg, 11.5-16 kg, 7–11.5 kg and 5-9 kg, respectively. Gestational weight gain was defined as the difference between the weight before delivery and the weight before pregnancy. Other potential related factors of LBW included in the interview were parity, antenatal care, maternal health status and essential information about the newborn.

The prospective case enrollment included information regarding delivery (e.g., gestational age and the mode of delivery), integrated information about the newborns, and information regarding antenatal care and medical conditions before, at and post-delivery (e.g., gestational hypertension, gestational diabetes mellitus and anemia during pregnancy). The information was collected from the obstetric records. The gestational age estimate that was recorded was assumed to represent the best available clinical estimate. Birth weight was measured using an infant electronic scale within 1 h of birth. In addition, the duplicate information about risk factors included in both the questionnaire and checklist were cross-checked to ensure data quality.

### Statistical analysis

All analyses were conducted using SPSS 22.0 (SPSS Inc., Chicago, IL, USA). All potentially relevant variables were first analyzed with a bivariate logistic regression to assess the associations between the preterm and term group: general demographic characteristics (age, marital status, level of education, economic status), pre-pregnancy BMI, parity, history of LBW, antenatal care, gestational hypertension and fetal sex. The criterion for selecting variables was set at a *p*-value less than 0.1 (90% level of significance) in the bivariate analysis. Second, to control for potential confounders, a multivariate logistic regression was performed. We used the *odds ratios* (ORs) and 95% confidence intervals (CIs) to make comparisons between the groups. The *p*-values of all the statistical analyses were two sided. The level of significance for the multivariate analysis was 95% (*P* < 0.05).

## Results

### Socio-demographic characteristics

The study criteria were met in 294 preterm LBW cases, 289 term LBW cases and 1381 controls. Table 1 shows the distribution of the socio-demographic characteristics. The majority of women were 19 to 34 years of age (80.3, 85.1 and 87.2% among the preterm LBW, term LBW and control groups, respectively). The majority of pre-pregnancy BMI were in the normal range (59.5, 49.8 and 63.0% among the preterm LBW, term LBW and control groups, respectively). Regarding economic status, the proportions of monthly income less than 3000Ұwere 35.0, 35.6 and 39.0% in the preterm LBW, term LBW and control groups, respectively. More than 90% of pregnant women were married and more than 90% had no history of LBW. The proportion of parity more than one was 37.4, 36.0 and 45.5% in the preterm LBW, term LBW and control groups, respectively. The frequency of antenatal care was most often 5–9 times or 10–14 times in all groups. In addition, 17.3, 13.1 and 3.1% of mothers in the preterm LBW, term LBW and control groups, respectively, had gestational hypertension.

**Table 1:** The association of socio-demographic variables with low birth weight

Variable	Reference	Total (*n* = 1964)	Normal birth weight (*n* = 1381)	Preterm low birth weight (*n* = 294)	Term low birth weight (*n* = 289)
		N (%)	N (%)	N (%)	N (%)
Age (years)	19–34	1686 (85.8)	1204 (87.2)	236 (80.3)	246 (85.1)
	≤18	36 (1.8)	18 (1.3)	5 (1.7)	13 (4.5) **
	≥35	242 (12.3)	159 (11.5)	53 (18.0) **	30 (10.4) *
Marital status	Married	1862 (94.8)	1326 (96.0)	272 (92.5)	264 (91.3)
	Unmarried or divorced	102 (5.2)	55 (4.0)	22 (7.5) **	25 (8.7) **
Education level	Primary or below	68 (3.5)	41 (3.0)	12 (4.1)	15 (5.2)
	Middle	635 (32.3)	418 (30.3)	109 (37.1)	108 (37.4)
	High	662 (33.7)	468 (33.9)	97 (33.0)	97 (33.6) *
	Junior college	347 (17.7)	269 (19.5)	39 (13.3) *	39 (13.5) **
	Undergraduate or beyond	252 (12.8)	185 (13.4)	37 (12.6)	30 (10.4) **
Economic status (Ұ/month)	≤3000	735 (37.4)	468 (33.9)	132 (44.9)	135 (46.7)
	3001–5000	744 (37.9)	538 (39.0)	103 (35.0) **	103 (35.6) **
	5001–7000	275 (14.0)	205 (14.8)	37 (12.6) **	33 (11.4) **
	> 7000	210 (10.7)	170 (12.3)	22 (7.5) **	18 (6.2) **
Pre-pregnancy BMI	18.5–23.9	1189 (60.5)	870 (63.0)	175 (59.5)	144 (49.8)
	< 18.5	573 (29.2)	364 (26.4)	88 (29.9) *	121 (41.9) **
	24–27.9	161 (8.2)	119 (8.6)	23 (7.8) *	19 (6.6)
	≥28	41 (2.1)	28 (2.0)	8 (2.7) *	5 (1.7)
Parity	1	1121 (57.1)	752 (54.5)	184 (62.6)	185 (64.0)
	>1	843 (42.9)	629 (45.5)	110 (37.4) **	104 (36.0) **
History of LBW	No	1889 (96.2)	1354 (98.0)	266 (90.5)	269 (93.1)
	Yes	75 (3.8)	27 (2.0)	28 (9.5) **	20 (6.9) **
Antenatal care	≤4	231 (11.8)	119 (8.6)	68 (23.1)	44 (15.2)
	5–9	914 (46.5)	582 (42.1)	190 (64.6) **	142 (49.1) **
	10–14	752 (38.3)	620 (44.9)	34 (11.6) **	98 (33.9) **
	≥15	67 (3.4)	60 (4.3)	2 (0.7) **	5 (1.7) **
Gestational hypertension	No	1832 (93.3)	1338 (96.9)	243 (82.7)	251 (86.9)
	Yes	132 (6.7)	43 (3.1)	51 (17.3) **	38 (13.1) **
Gestational diabetes mellitus	No	1702 (86.7)	1208 (87.5)	248 (84.4)	246 (85.1)
	Yes	262 (13.3)	173 (12.5)	46 (15.6)	43 (14.9)
Anemia during pregnancy	No	1548 (78.8)	1082 (78.3)	240 (81.6)	226 (78.2)
	Yes	416 (21.2)	299 (21.7)	54 (18.4)	63 (21.8)

Note: In the analysis, the preterm and term LBW groups were compared with the normal weight group; *p < 0.1; **p < 0.05

### Determinants of preterm and term LBW

The distribution of lifestyle factors in the cases and control are shown in Table 2. In this survey, 99.6% of the pregnant women did not smoke; therefore, smoking was not included in our analysis. However, the proportion of pregnant women exposed to passive smoking was 38.4% in the term LBW group, in contrast with 33.1% in the control group. Drinking rates were similar in the three groups (3.1, 2.7 and 1.6% in the preterm LBW, term LBW and control groups, respectively). In the preterm LBW group, the proportion of mothers who engaged in physical activity 1–3 and ≥ 4 times per week was 32.3 and 39.8%, in contrast with the 34.5 and 45.9% in the control group. Regarding gestational weight gain, the proportions of insufficient gestational weight gain in the preterm LBW, term LBW and control groups were 51.4, 49.1 and 31.0%, respectively. In addition, the rates of abstinence from coffee or tea in the three groups were similar (> 80%).

**Table 2:** Description of the lifestyle characteristics and their association with low birth weight

Variable	Reference	Total (*n* = 1964)	Normal birth weight (*n* = 1381)	Preterm low birth weight (*n* = 294)	Term low birth weight (*n* = 289)
		N (%)	N (%)	N (%)	N (%)
Passive smoking	No	1289 (65.6)	924 (66.9)	187 (63.6)	178 (61.6)
	Yes	675 (34.4)	457 (33.1)	107 (36.4)	111 (38.4) **
Drinking	No	1925 (98.0)	1359 (98.4)	286 (97.3)	280 (96.9)
	Yes	39 (2.0)	22 (1.6)	8 (2.7)	9 (3.1) *
Coffee or tea consumption (/week)	No	1667 (84.9)	1156 (83.7)	260 (88.4)	251 (86.9)
	1–3	257 (13.1)	194 (14.0)	30 (10.2)	33 (11.4)
	≥4	40 (2.0)	31 (2.2)	4 (1.4)	5 (1.7)
Physical activity (/week)	No	419 (21.3)	271 (19.6)	82 (27.9)	66 (22.8)
	1–3	672 (34.2)	476 (34.5)	95 (32.3) **	101 (34.9)
	≥4	873 (44.5)	634 (45.9)	117 (39.8) **	122 (42.2)
Gestational gain	Normal	770 (39.2)	570 (41.3)	86 (29.3)	114 (39.4)
	Less	721 (36.7)	428 (31.0)	151 (51.4) **	142 (49.1) **
	More	473 (24.1)	383 (27.7)	57 (19.4)	33 (11.4) **

Note: In the analysis, the preterm and term LBW groups were compared with the normal weight group;

*p < 0.1; **p < 0.05

### Bivariate and multivariate analysis

The bivariate and multivariate analysis of socio-demographic characteristics in the preterm LBW and control groups revealed that the LBW status was significantly associated with age more than 35 years (cOR = 1.713, aOR = 2.486), parity more than one (cOR = 0.330, aOR = 0.273), history of LBW (cOR = 5.279, aOR = 5.517), gestational hypertension (cOR = 6.377, aOR = 9.062) and antenatal care. In the term LBW and control groups, the LBW status was significantly associated with pre-pregnancy BMI less than 18.5 (cOR = 2.008, aOR = 1.718), parity more than one (cOR = 0.535, aOR = 0.506), history of LBW (cOR = 3.728, aOR = 4.087), gestational hypertension (cOR = 4.600, aOR = 6.598) and antenatal care.

In the bivariate analysis, physical activity and gestational weight gain were significantly associated with preterm LBW. However, in the bivariate analysis, passive smoking and gestational weight gain were significantly associated with term LBW. The results of multivariate analysis are summarized in Tables 3 and 4. After other variables were controlled, physical activity had a positive association with preterm LBW, regardless of frequency, compared with no physical activity (1–3/week: aOR = 0.584, 95%CI = 0.394–0.867; ≥ 4/week: aOR = 0.516, 95%CI = 0.355–0.752, respectively). Women who had insufficient gestational weight gain were more likely to deliver a preterm LBW baby (aOR = 2.272, 95%CI = 1.626–3.176). Moreover, age ≥ 35 years was significantly associated with preterm LBW. As shown in Table 4, after relevant variables were controlled, passive smoking was found to be a positive factor for term LBW. Women who experienced passive smoking during pregnancy had a higher probability of having term LBW babies (aOR = 1.404, 95%CI = 1.057–1.864). Insufficient gestational weight gain had a significant association with term LBW (aOR = 1.484 95%CI = 1.103–1.998), and excessive gestational weight gain had a positive association with term LBW (aOR = 0.369, 95%CI = 0.236–0.577). These findings suggest that a pre-pregnancy BMI < 18.5 was significantly associated with term LBW. In addition, as shown in Tables 3 and 4, parity, history of LBW, antenatal care and gestational hypertension were significantly associated with preterm and term LBW.

**Table 3:** Multivariate logistic analyses showing the outcomes associated with preterm low birth weight

Variable		Reference	P	Crude OR	Adjusted OR^a^	95%CI
Physical activity (/week)	1–3	No	0.008	0.630	***0.584***	***0.394–0.867***
	≥4		0.001	0.601	***0.516***	***0.355–0.752***
Gestational gain	Less	Normal	< 0.001	2.338	***2.272***	***1.626–3.176***
	More		0.329	0.986	0.811	0.532–1.236
Age (years)	≤18	19–34	0.481	1.418	0.551	0.161–1.882
	≥35		< 0.001	1.713	***2.486***	***1.599–3.865***
Parity	>1	1	< 0.001	0.330	***0.273***	***0.192–0.389***
History of LBW	Yes	No	< 0.001	5.279	***5.517***	***2.831–10.750***
Antenatal care	5–9	≤4	0.010	0.553	***0.593***	***0.398–0.884***
	10–14		< 0.001	0.093	***0.083***	***0.049–0.141***
	≥15		< 0.001	0.056	***0.044***	***0.010–0.198***
Gestational hypertension	Yes	No	< 0.001	6.377	***9.062***	***5.327–15.415***

Note: *OR* odds ratio; *CI* confidence interval; *LBW* low birth weight Control: normal weight term baby

^a^Variables in the logistic regression include maternal characteristics, pre-pregnancy BMI, parity, history of LBW, antenatal care, fetal sex and gestational hypertension

**Table 4:** Multivariate logistic analyses showing the outcomes associated with term low birth weight

Variable		Reference	P	Crude OR	Adjusted OR^a^	95%CI
Passive smoking	Yes	No	0.019	1.356	***1.404***	***1.057–1.864***
Gestational gain	Less	Normal	0.009	1.659	***1.484***	***1.103–1.998***
	More		< 0.001	0.431	***0.369***	***0.236–0.577***
Economic status (Ұ/month)	3001–5000	≤3000	0.032	0.669	***0.715***	***0.526–0.971***
	5001–7000		0.104	0.576	0.687	0.437–1.080
	> 7000		0.026	0.391	0.526	0.299–0.926
Pre-pregnancy BMI	< 18.5	18.5–23.9	< 0.001	2.008	***1.718***	***1.278–2.310***
	24–27.9		0.826	0.965	1.065	0.607–1.868
	≥28		0.569	1.079	1.409	0.433–4.579
Parity	>1	1	< 0.001	0.535	***0.506***	***0.368–0.695***
History of LBW	Yes	No	< 0.001	3.728	***4.087***	***2.070–8.069***
Antenatal care	5–9	≤4	0.344	0.664	***0.808***	***0.519–1.257***
	10–14		0.014	0.425	***0.557***	***0.349–0.889***
	≥15		0.024	0.222	***0.305***	***0.108–0.858***
Gestational hypertension	Yes	No	< 0.001	4.600	***6.598***	***3.915–11.122***

note: *OR* odds ratio; *CI* confidence interval; *LBW* low birth weight; *BMI* body mass index; *Control* normal weight term baby

^a^Variables in the logistic regression include maternal characteristics, pre-pregnancy BMI, parity, history of LBW, antenatal care, fetal sex, and gestational hypertension

## Discussion

These findings suggest that maternal physical activity during pregnancy has a positive association with preterm LBW but not term LBW. Pregnant women who were exposed to smoking during pregnancy had increased odds of having term LBW babies. In addition, insufficient gestational weight gain had a significant association with preterm and term LBW and excessive gestational weight gain was only significantly associated with term LBW.

This study showed that socio-demographic characteristics could affect the weight of newborns. Parity and antenatal care were both positively associated with LBW, in accordance with previous findings [33, 34]. Adequate antenatal care may help women identify risks early in pregnancy and allow timely intervention; however, studies from Japan and Sweden also suggest that higher parity with shorter pregnancy intervals could increase the risk of LBW [35, 36]. Gestational hypertension was shown by Buchbinder to be a clear risk factor for LBW [37], in agreement with these findings. In addition, this study found that pregnant over 35 had a higher risk of delivering preterm LBW babies. A possible explanation for this association is that age may influence changes in the uterine vasculature, leading to higher rate of LBW infants in women over 35 [38]. In the term LBW group, pre-pregnancy BMI was a significant factor associated with LBW, as women with a pre-pregnancy BMI < 18.5 were more likely to have term LBW babies. A study conducted in Japan showed that an underweight pre-pregnancy BMI was independently associated with LBW in full-term babies [39].

In the preterm LBW group, this study suggested that moderate intensity physical activity may decrease rates of preterm LBW. Similarly, previous studies suggested that appropriate physical activity was a protective factor for LBW [14, 40]. The potential explanations for this findings was that the effects of moderate intensity physical activity during pregnancy can extend gestational age to reduce the risk of having LBW babies and can influence endocrine regulation of fetal growth and promote an increase in the ratio of muscle to adipose tissue mass [29]. However, in previous studies, due to the variability and types of physical activity and potential recall bias, the association between physical activity and preterm LBW might be hard to accurately analyze. Regarding the types of activity, Leiferman and Evenson suggested a protective effect of regular leisure time physical activity [41]. Population studies have suggested that regular, low to moderate intensity physical activity reduces the risk of babies born at the extreme ends of the birth weight range [42]. In addition, constant physical exercise in pregnant women reduces the risk of LBW outcomes [40]. Meanwhile, there have been studies showing that physical activity was not associated with LBW. In Sweden, the study by Hegaard showed that pregnant women who practiced sports or leisure-time physical activity gave birth to infants with a similar weight as inactive women [43].

In the term LBW group, data in the study confirmed that passive smoking was associated with LBW. A previous study suggested that passive smoking was associated with LBW [44]. A study in the Netherlands suggested that passive smoking during pregnancy was only associated with term LBW [45]. In addition, the meta-analysis by Salmasi suggested that passive smoking caused infants to weigh less, with a trend towards an increased incidence of LBW [46],Martin et al. also reported the relationship only occurred in term (≥37 weeks) deliveries [47]. The reduced oxygenation of the fetus secondary to carbon monoxide as well as nicotine-related vasoconstriction may result in decreased uterine and placental blood flow [44, 48]. Maternal exposure to passive smoking appears to relate only to growth restriction [49] and not to preterm delivery [45]. These findings suggested that exposure to environmental tobacco smoke in non-smoking pregnant women was associated with a lower birth-weight, rather than LBW; the reasons for the difference might include the method and place of measurement [50].

The study by Scholl suggested that insufficient gestational weight gain was associated with inadequate nutrient intake [51], which was similar to our result. Moreover, previous studies suggested that lack of consumption of nutritious food during pregnancy was significantly associated with LBW babies [24] and that the reason for insufficient gestational weight gain was, in part, due to low energy intake [52]. However, in the term LBW group, excessive gestational weight gain decreased the risk of delivering term LBW babies. Previous evidence also suggested that term birth weight increases dramatically with higher weight gain during pregnancy [53], in the USA, women who gained > 25 lbs. (above IOM recommendations) had a lower probability for LBW [54]. The explanation may be that hyperglycemia stimulates insulin which serves as a growth hormone for the fetus during pregnancy and excessive weight gain may result in an overproduction of insulin. However, women who gain excessive amounts of weight might consume too many calories during pregnancy, which, in turn, can further accelerate fetal growth, increasing the risk for other complications, such as preeclampsia, cesarean section, and macrosomia [55–57].

This study has several strengths but also some limitations. Its main strength is that the case group was divided into a preterm and a term group. These groups were propitious for exploring the real association between lifestyle factors and preterm and term LBW and for providing guidance for the development of appropriate and sustainable interventions to improve maternal health. Another strength is that we considered confounders including economic status, and education level, which may influence the association with birth weight.

One limitation is that participants were not matched due to logistic constraints of recruitment in 14 hospitals. However, in most previous studies subjects were not matched [25, 58]. Another limitation is that lifestyle factors were assessed through a retrospective self-reported questionnaire, which may have introduced recall bias and the possibility of exposure misclassification associated with the evaluation of multiple routes of exposure.

## Recommendation

This study suggests that pregnant women without exercise contraindications should perform regular appropriate physical activity to improve their health status and pregnancy outcomes. Pregnant women should be aware of the dangers of passive smoking and be provided strategies to decrease exposure. As women are particularly vulnerable to the impacts of passive smoking in public places, research and health programs have focused on mothers and their LBW newborns [59]. These findings recommend that women achieve only moderate weight gain in pregnancy. Adequate intake of nutrients, a balanced diet and professional guidance may help to optimize gestational weight.

## Conclusion

This study was conducted to investigate the impact of lifestyle factors on LBW in preterm and term births after controlling for relevant confounders. Physical activity may be associated with a reduced risk of preterm LBW and pregnant women who experience passive smoking have an increased risk of delivering term LBW babies. Moreover, insufficient gestational weight gain had a significant association with preterm and term LBW.

## Data Availability

We declare that materials described in the manuscript, including all relevant raw data, will be freely available to any scientist wishing to use them for non-commercial purposes, without breaching participant confidentiality.

## Abbreviations

LBW: Low birth weight
BMI: Body mass index
WHO: World Health Organization
UNICEF: United Nations Children’s Fund
